# Validation of the Malay-Version of Recovery Knowledge Inventory among mental health providers in Malaysia

**DOI:** 10.3389/fpsyt.2023.1078423

**Published:** 2023-06-13

**Authors:** Stella Jane Lakshman, Tuti Iryani Mohd Daud, Marhani Midin, Farah Ahmad, Kezia Tommy

**Affiliations:** ^1^Department of Psychiatry, Faculty of Medicine, Universiti Kebangsaan Malaysia, Kuala Lumpur, Malaysia; ^2^Hospital Canselor Tuanku Muhriz, Jalan Yaacob Latif, Bandar Tun Razak, Kuala Lumpur, Malaysia; ^3^Department of Psychiatry, Hospital Selayang, Batu Caves, Selangor, Malaysia; ^4^Hospital Bahagia Ulu Kinta, Perak, Malaysia; ^5^Faculty of Business and Economics, University Malaya, Kuala Lumpur, Malaysia

**Keywords:** Malaysia, validation, confirmatory factor analysis, Recovery Knowledge Inventory, recovery scale

## Abstract

**Background:**

The Recovery Knowledge Inventory (RKI) is a widely used self-report instrument that assesses recovery-oriented knowledge among mental health professionals. The purpose of this study is to translate the RKI into the Malay language (RKI-M) and to examine its psychometric properties among Malaysian health care workers.

**Methods:**

A cross-sectional study involving 143 participants was conducted at an urban teaching hospital, an urban government hospital, and a rural government hospital. Following the translation of the RKI, its internal reliability was determined using Cronbach’s alpha. Construct validity was also determined using confirmatory factor analysis.

**Results:**

The Malay-Version RKI (RKI-M) has good internal reliability with a Cronbach’s alpha of 0.83. However, the Malay-version RKI failed to replicate the original four-factor structure. The final model only achieved the best model fit after the removal of 9 items with two-factor loadings: (GFI = 0.92; AGFI = 0 0.87; CFI = 0.91; RMSEA = 0.074).

**Conclusion:**

The 20-item RKI-M is reliable but has poor construct validity. However, the modified 11-item Malay-version RKI is a more reliable measure as it has good construct validity, with room for future studies to examine the psychometric properties of the modified 11-item RKI among mental health care workers. More training on recovery knowledge should be done, and a simple worded questionnaire should be developed in keeping with local practitioners.

## 1. Introduction

Recovery in mental illness is an evolving concept that has come a long way since the 1990s. The process of changing one’s attitudes, values, feelings, goals, abilities, and roles during recovery is deeply personal and it’s a way to manage mental illness while still leading a happy, purposeful life (1). Currently, an increasing number of comprehensive frameworks have attempted to secure the various components of personal recovery (2, 3). One of the most well-known attempts to successfully integrate the numerous recovery constructs already in existence is the CHIME framework. The acronym “CHIME” stands for the model’s five recovery processes (3) (i.e., connectedness, hope, identity, meaningfulness, and empowerment). The “C” stands for connectedness, and while less obviously individualistic than Anthony's (1) framework definition, it still exhibits many of the same traits as earlier conceptions of recovery.

Measuring knowledge of recovery is vital in ensuring practices are in keeping with the current understanding of the recovery-oriented approach. It is essential that a valid and reliable tool be in place to evaluate mental health workers’ recovery knowledge. Such a tool should be able to gauge the level of knowledge and help improve psychiatric services. The Recovery Knowledge Inventory (RKI) was developed by Bedregal et al. (4) to assess the knowledge on mental health illness and the recovery approach among mental health staff. RKI seeks information on roles and responsibilities in recovery (4). It is used to understand the nonlinear process and roles of self-definitions in recovery and the expectation of recovery (2). The English-version RKI has 20 items and a four-factor structure based on the following four items: (i) roles and responsibilities in recovery, (ii) non-linearity of the recovery process, (iii) roles of self-definition and peers in recovery, and (iv) expectations about recovery. The Cronbach’s α coefficients for each domain were 0.81, 0.70, 0.63, and 0.47 (4). The Higher scores indicate more knowledge and positive attitudes toward concept of Recovery (4).

In recent times, research on recovery has often been qualitative rather than empirical. Recovery is usually labeled as a non-linear journey that can be affected by multifaceted factors. The importance of hope and optimism, respecting the knowledge of the service user, valuing diversity, and allowing for risk-taking behaviors are common themes for service delivery that replicate the ideologies of the recovery movement (5). However, for mental health professionals to use this treatment ideology, a deeper comprehension of the ideas of attitude change and recovery is necessary (6).

Malaysia, as a developing nation, is made up of a multi-cultural and multi-lingual community, so it is paramount to develop a validated tool that measures the knowledge of recovery in its national language, Bahasa Malaysia, which is widely used in the country and can be easily understood. This research provides a significant chance to advance our understanding of the recovery approach. At the time of literature review, there were no specific study on recovery-based knowledge and the extent of it being part of the local practices. This study was done to first have a validated objective tool to understand how far our local mental health professionals have a grasp on this model. It is believed that most psychiatrists in Malaysia currently limit their practises to functional and symptom relief and, at times, have some recovery-based practises without realizing it. Furthermore, by using a validated tool objectively, such an effort will be instrumental in shaping the future of mental health services in Malaysia. We predict that the Bahasa Malaysia (RKI-M) will have a satisfactory factorial validity and reliability. It is important to translate and validate the RKI to Bahasa Malaysia to encourage more studies, not only among mental health workers but also among allied health. This study was done with two questions in mind: (i) What is the internal consistency of the RKI-M in a sample of Malaysian Mental Health care workers? and (ii) Will the RKI be able to establish construct validity with the Malay Version of the Recovery Knowledge Inventory in a sample of Malaysian mental health care workers? The main objective of this study is to translate the RKI into Bahasa Malaysia and to examine the psychometric properties of the RKI-M.

## 2. Methods

### 2.1. Study design

A cross-sectional study was conducted in the Psychiatry department of three different hospitals, specifically Hospital Canselor Tuanku Muhriz (HCTM), Hospital Tuanku Jaafar Seremban (HTJS), and Hospital Tuanku Ampuan Najihah (HTAN) Kuala Pilah in March 2021. Permission from both the UKM Ethics committee and from the Director-General of Health Malaysia were obtained prior to publication (NMRR-20-301257,590).

HCTM, a teaching center in a university setting, was chosen together with HTJS, a tertiary hospital under the administration of Ministry of Health Malaysia. Both centers are in an urban setting and have both inpatient and community care services. HTAN, which is situated in the rural area of Negeri Sembilan, despite not having a ward setting, has a dedicated team of community mental health care. Purposive sampling was used to include health care workers from rural, urban, and teaching hospitals to obtain a more heterogeneous sample population.

### 2.2. Development of the Malay Version RKI (RKI-M)

The Recovery Knowledge Inventory has 20 questions comprising four scales: (I) Roles and Responsibilities, (II) Non-Linearity of the Recovery Process, (III) Roles of Self-definition and Peers, and (IV) Expectations Regarding Recovery. This tool uses a 5-point Likert-type scale, which are 1 (Strongly disagree), 2 (Disagree), 3 (Not sure), 4 (Agree), and 5 (Strongly agree). Higher scores indicate having greater knowledge and a more positive attitude toward the concept of recovery. Fifteen items were scored inversely to minimize the influence of social desirability (4). The RKI was translated into the Malay language following permission from the original developer. The translation was based on guidelines for translating and adapting psychometric scales by Gudmundsson (7). First, the English version RKI was translated into Malay independently by two bilingual authors, a psychiatrist, and a linguist. Both translations were then compared and combined to become the Malay-version RKI (8). Then, a different pair of translators (a linguist and a psychiatrist), also bilingual, back-translated the Malay version of RKI-M independently. The translators were first briefed on the target population of the questionnaires. Following this step, the researchers cross-checked the back translation with the original questionnaire. Editing and revision of the translated version were then done to ensure literal and conceptual equivalence between the original RKI and the Malay-version RKI. Next, a pilot study was conducted on 30 participants comprising medical students and house officers currently in their psychiatric rotation (9–11). The participants were enquired about their ease in understanding the questionnaire, and their comments were used to make changes to the RKI-M.

### 2.3. Participants

One hundred and forty-three participants were recruited through purposive sampling from the three hospitals mentioned above. In keeping with the standard practice applied for factor analysis when the given number of items is 20, as a rule of thumb, a minimum of 5–10 samples is needed per item, which computes to 100 minima to 200 maximum samples (12). In estimating 20% of the non-response rate being (100 × 0.2 = 20/200 × 0.2 = 40), the total sample required will thus be 120–240. For the Confirmatory Factor Analysis (CFA), more than 3 items per construct plus 0.45–0.55 communality was used (13). The inclusion criteria included all mental health service providers such as registered staff nurses, medical assistants, occupational therapists, psychologists, psychiatrists, and medical officers who have been serving in the psychiatry department for more than 1 year. The exclusion criteria were non-mental health workers such as clerks, hospital attendants, and incomplete questionnaires.

**Figure 1:**
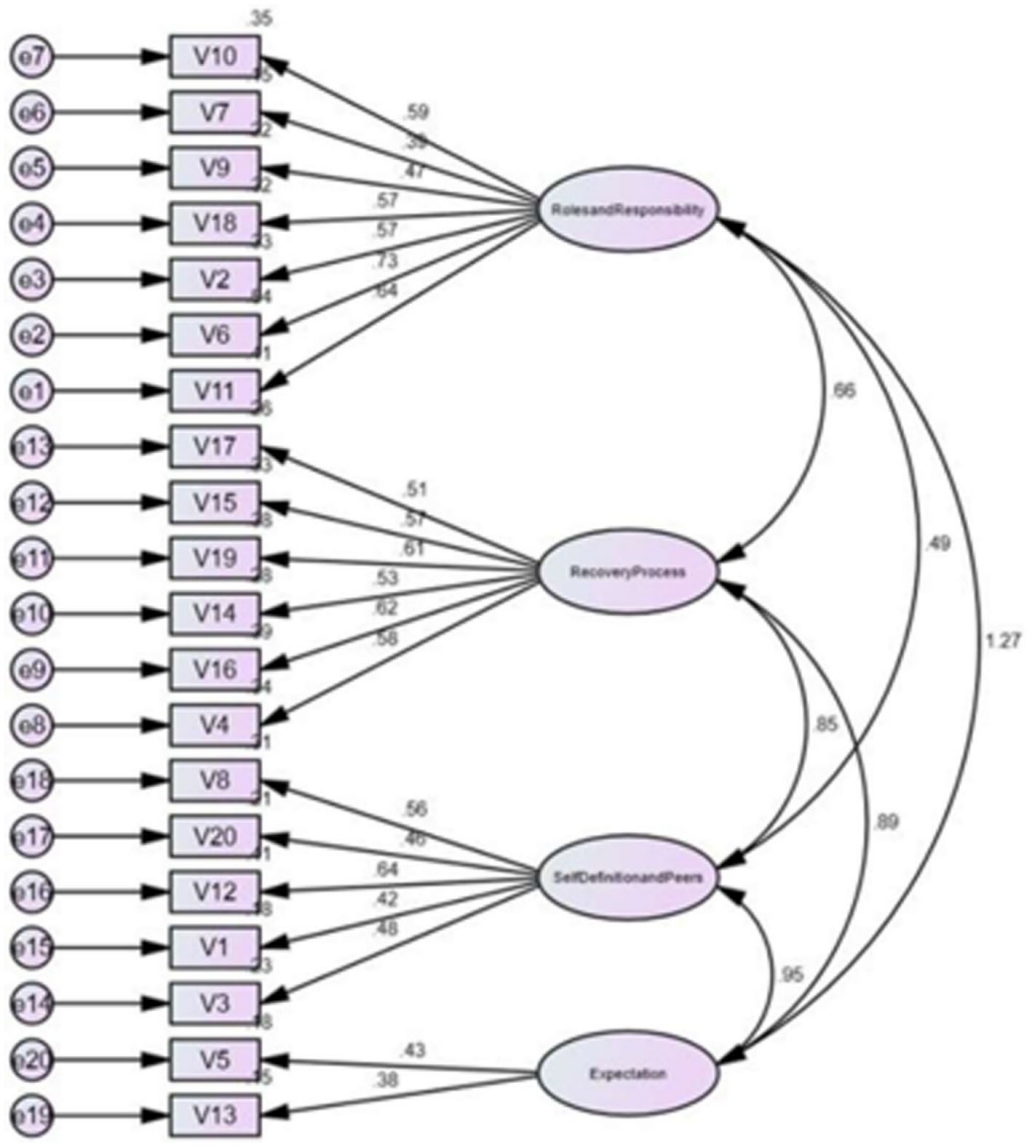
Confirmatory factor analysis of four factors.

**Figure 2:**
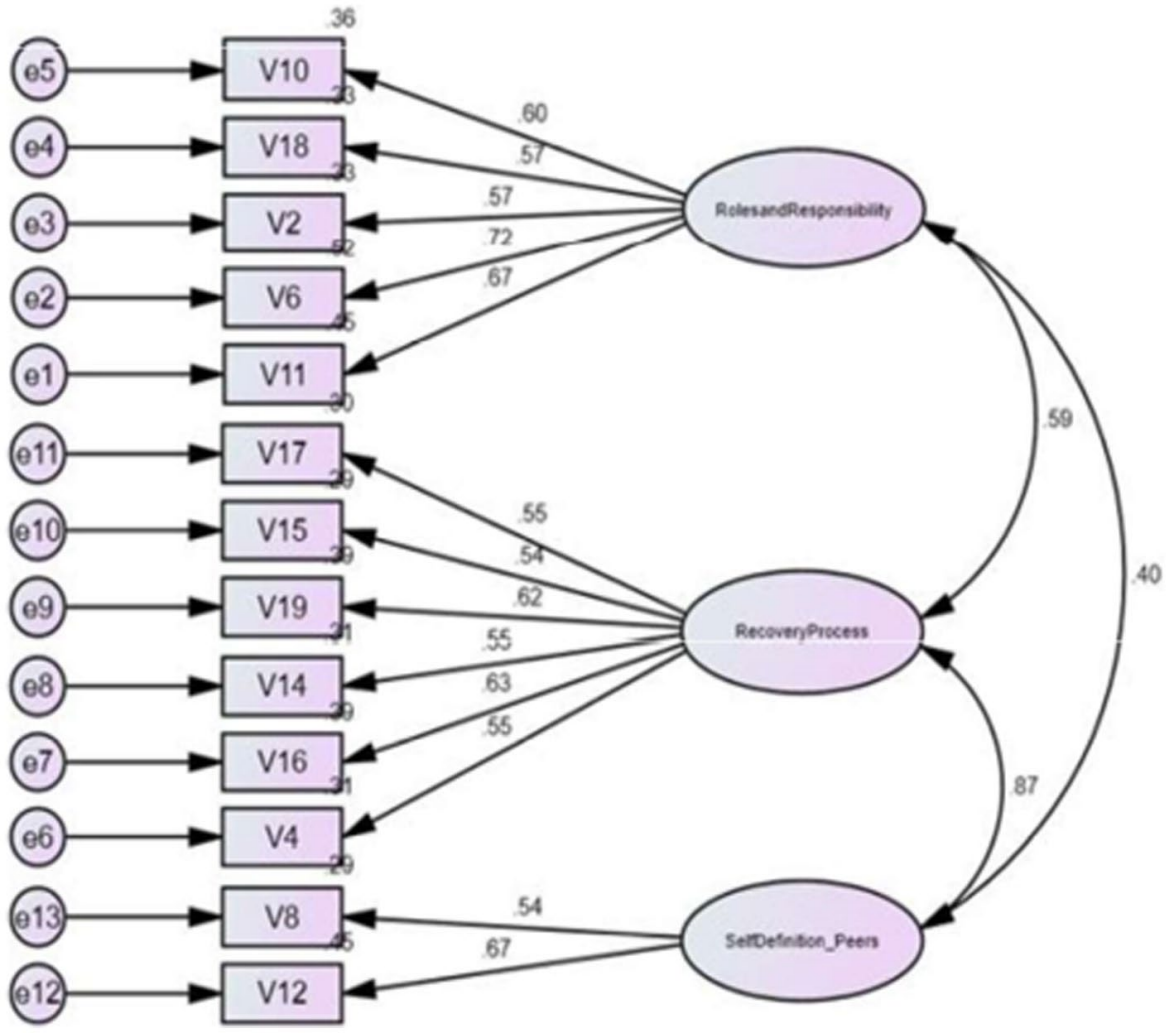
Confirmatory factor analysis of three factors.

**Figure 3:**
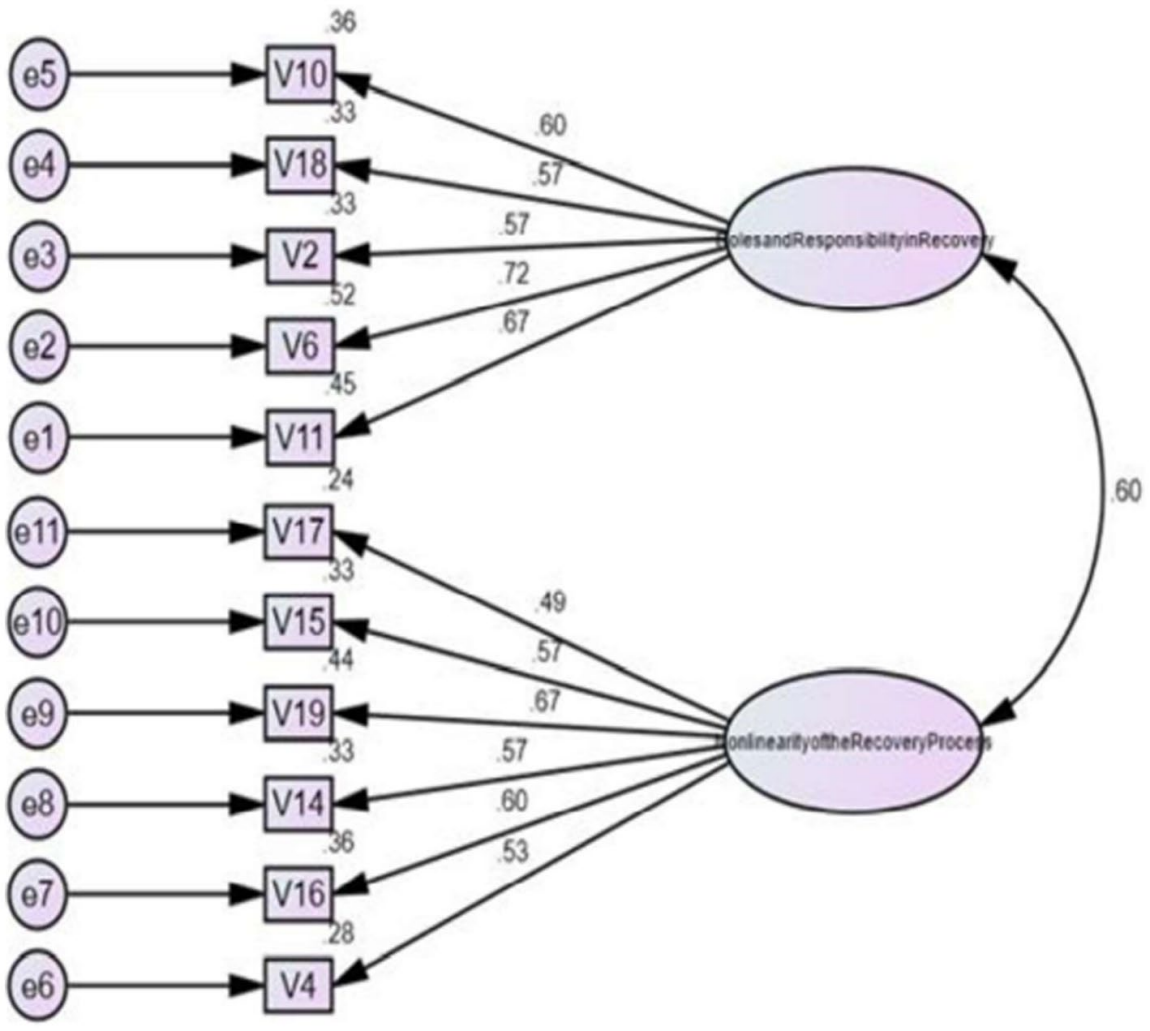
Confirmatory factor analysis of two factors.

### 2.4. Procedure

Data collection was carried out both onsite and online during the COVID-19 pandemic in March 2021. The study information sheet that had the objective, the purpose, the inclusion, exclusion criteria along with the consent. The translated version of the RKI-M questionnaire was distributed to all mental health care workers at the said hospitals. In view of the social distancing practices, participants were given the option to fill up an online Google or a hardcopy form. The same questions as the hardcopy were used and the Google Forms link was disseminated *via* the Department’s social media platform. The link was also displayed and shared during all Department’s teachings and meetings. Participants were reminded to only choose one mode of answering the questionnaire to avoid double sampling. The participants had to give their consent before they could proceed further. A physical drop box was provided at a designated place for the manual forms to be recollected. All participants were kept anonymous as no email or identification numbers were obtained.

### 2.5. Data analysis

Using SPSS software, all statistical analyses were performed. AMOS software was used for CFA. Given that the original study had performed an Exploratory Factor Analysis (EFA), following a series of discussion, the authors decided that a Confirmatory Factor Analysis (CFA) would be more beneficial to test the fitness of the data for this study (14). The study was conducted using the estimation procedure called Maximum Likelihood Confirmatory Factorial Analysis (15). The factor structure of RKI-M was tested using CFA against the four factors identified by the authors of the original RKI (4). The following fit indices were used for evaluating the model fit: (1) the goodness-of-fit index (GFI > 0.90); (2) the adjusted goodness-of-fit index (AGFI > 0.80); (3) the Comparative fit index (CFI > 0.90); (4) the root mean square error of approximation (RMSEA < 0.08); and (5) CMIN/df (<5.0 usually indicates a good fit) (16–18). It is considered acceptable if the factor loading is >0.30. Anything less than this value would imply a poor relationship between the variables (19). Internal consistency was measured using Cronbach’s alpha, where a value of 0.7 or more is considered acceptable (20). For the structural equation model, a value of *p* < 0.5 is considered statistically significant (21) (Figures 1–3).

## 3. Results

### 3.1. Characteristics of participants

We obtained a total of 143 questionnaires for analysis. A total of 97 participants were from HCTM but only 58 completed the questionnaire (a 60% response rate). From the HTJS tertiary hospital, a total of 95 participants were eligible but only 65 responded (a 68% response rate). HTAN, a rural hospital, had 20 eligible participants and all of them responded (a 100% response rate). The participants’ sociodemographic and occupational characteristics are shown in Table 1. Most of the participants were female, and the mean age was 36 years (±6.75). The mean length of experience in Psychiatric services was 11 years (±6.15). The largest occupational group was nurses, comprising about a quarter of the respondents, followed by medical officers. Ethnically, the majority were Malay (75%), followed by Indian (11.9%), and Chinese (8.4%).

**Table 1:** Characteristics of the study participants (*n* = 143).

Variables		
Sex, *N* (%)		
Male	46	(32.2)
Female	97	(67.8)
Age (years), mean (±SD)	36.04	(6.7)
Duration working in current facility, mean in years (±SD)	11.2	(6.2)
Duration of working in psychiatric services: mean in years (±SD)	8.5	(7.5)
Ethnicity, *N* (%)		
Malay	108	(75.5)
Chinese	12	(8.4)
Indian	17	(11.9)
Others	16	(4.2)
Occupation, *N* (%)		
Psychiatrist	18	(12.6)
Psychologist	5	(3.5)
Staff nurse	40	(28.0)
Medical assistant	16	(11.2)
Psychiatry trainee	16	(11.2)
Psychiatric medical officer	41	(28.7)
Occupational therapist	7	(4.9)
Study location, *N* (%)		
Teaching hospital (Urban)	58	(40.6)
Ministry of health hospital (Urban)	65	(45.5)
Ministry of health hospital (Rural)	20	(14.0)

Table 2 shows the total mean score between the three different hospitals based on the 11-item RKI-M. HCTM scored a lower mean compared to HTJS while HTAN scored higher, indicating that the latter two hospitals had lesser knowledge of recovery, as their answers were not in keeping with the tenets of the recovery approach. This result could be explained by the fact that HCTM is more academic and has a responsibility to disseminate recent evidence and knowledge. Almost all the staff, doctors, and allied mental health workers in HCTM have had specific training or were currently being trained in psychiatry. The other two Kementerian Kesihatan Malaysia (KKM) hospitals are more service-oriented and are not purely engaged in psychiatry academia and may consist of more lay personnel who might be more heterogenous in knowledge and experience in psychiatry. This data showed that the urban or rural areas did not really matter as much as the setting of the hospital.

**Table 2:** Total mean score between the 3 different hospitals based on the 11-item RKI-M (*N* = 143).

Study location	Teaching hospital (Urban)			Ministry of health hospital (Urban)			Ministry of health hospital (Rural)		
	Mean	*N*	SD	Mean	*N*	SD	Mean	*N*	SD
Q1	2.93	58	1.18	3.28	65	1.34	3.70	20	1.26
Q2	4.34	58	0.61	4.48	65	0.59	4.45	20	0.51
Q3	2.03	58	0.77	2.51	65	1.15	2.70	20	1.30
Q4	3.12	58	1.01	3.18	65	1.22	3.45	20	1.28
Q5	3.05	58	1.23	3.17	65	1.40	3.30	20	1.26
Q6	3.64	58	0.79	3.72	65	0.96	4.10	20	1.07
Q7	3.53	58	0.98	4.12	65	0.80	3.95	20	1.10
Q8	4.17	58	0.65	4.29	65	0.68	4.40	20	0.60
Q9	4.34	58	0.51	4.34	65	0.62	4.30	20	0.57
Q10	3.50	58	0.92	3.60	65	1.00	3.65	20	1.14
Q11	4.28	58	0.70	4.37	65	0.67	4.60	20	0.60

### 3.2. Reliability of RKI-M

The Cronbach’s alpha coefficient for the 20-item RKI-M was 0.821. After the removal of the 9 items mentioned earlier, the Cronbach’s alpha coefficient became 0. 803. Factor 1 was 0.758, Factor 2 was 0.725, and Factor 3 was 0.533. Factor 3 had poor internal consistency and was not reliable. The other factors had acceptable internal consistency and were reliable.

### 3.3. Validity of RKI-M

#### 3.3.1. Confirmatory factorial analysis

The mean total of the 20-item RKI score was 76.1 (SD = 18.0; range: 28–100) (Table 3). Based on the original study by Bedregal et al. (4), we matched the items from RKI-M with the original RKI. The 20-item RKI-M did not yield satisfactory results with GFI = 0.82; AGFI = 0.77; CFI = 0.79; RMSEA = 0.082 (Table 4). We initially removed 7 items based on items with a factor loading of less than 0.3–0.4. Factors were reduced to 3. Because of the poor reliability of Factor 3, we proceeded to reduce the number of factors to 2. Hence, we removed a total of 9 items. The deleted items were items 1, 3, 5, 7, 8, 9, 12, 13, and 20. The two factors retained were roles and responsibilities in recovery (Factor 1) and the non-linearity of the recovery process (Factor 2). CFA based on the two-factor structure suggested a good fit to the data and satisfied three out of the four criteria needed to reach the recommended standards (GFI = 0.92; AGFI = 0 0.87; CFI = 0.91; RMSEA = 0.074; Table 5). Table 6 shows the mean comparison based on the different types of occupation. The data shows differences in the mean total for RKI-M in between all types of occupation. A one-way ANOVA revealed that there was a statistically significant difference in mean RKI score between groups (*F*(3, 139) = 12.762, *p* < 0.01). Nurses and medical assistants appear to have better recovery knowledge compared to psychiatrists and medical officers who scored on the lower side.

**Table 3:** Descriptive statistics for the 20-item RKI-M (*N* = 143).

No.	Item	Theoretical domain^a^	Mean	SD	Min.	Max.
1	The concept of recovery is equally relevant to all phases of treatment	Recovery readiness	4.66	0.70	1	5
2	People receiving psychiatric/substance abuse treatment are unlikely to be able to decide their own treatment and rehabilitation goals	Self determination	3.21^b^	1.28	1	5
3	All professionals should encourage clients to take risks in the pursuit of recovery	Risk taking	3.88	0.92	2	5
4	Symptom management is the first step toward recovery from mental illness/substance abuse	Managing symptoms	4.42^b^	0.59	2	5
5	Not everyone is capable of actively participating in the recovery process	Recovery readiness	3.78^b^	0.97	2	5
6	People with mental illness/substance abuse should not be burdened with the responsibilities of everyday life	Citizenship	2.34^b^	1.06	1	5
7	Recovery in serious mental illness/substance abuse is achieved by following a prescribed set of procedures	Individual process	4.08^b^	0.84	1	5
8	The pursuit of hobbies and leisure activities is important for recovery	Involvement in meaningful activities	4.48	0.66	2	5
9	It is the responsibility of professionals to protect their clients against possible failures and disappointments	Risk taking	3.39^b^	1.08	1	5
10	Only people who are clinically stable should be involved in making decisions about their care	Self determination	3.20^b^	1.15	1	5
11	Recovery is not as relevant for those who are actively psychotic or abusing substances	Recovery readiness	3.14^b^	1.31	1	5
12	Defining who one is, apart from his/her illness/condition, is an essential component of recovery	Redefining self	4.18	0.73	2	5
13	It is often harmful to have too high of expectations for clients	Hope	3.50^b^	1.03	1	5
14	There is little that professionals can do to help a person recover if he/she is not ready to accept his/her illness/condition or need for treatment	Incorporating illness	3.74^b^	0.92	2	5
15	Recovery is characterized by a person making gradual steps forward without major steps back	Non-linear progress	3.86^b^	0.95	1	5
16	Symptom reduction is an essential component of recovery	Managing symptoms	4.30^b^	0.66	2	5
17	Expectations and hope for recovery should be adjusted according to the severity of a person’s illness/condition	Hope	4.34^b^	0.57	3	5
18	The idea of recovery is most relevant for those people who have completed, or are close to completing, active treatment	Recovery readiness	3.57^b^	0.98	1	5
19	The more a person complies with treatment, the more likely he/she is to recover	Services are not enough	4.36^b^	0.68	1	5
20	Other people who have a serious mental illness or are recovering from substance abuse can be instrumental to a person’s recovery as mental health professionals	Supportive others	3.92	0.94	1	5

a The theoretical domain is based on 20 items from the original RKI (4).

b Item scores were inversed before calculating the mean and standard deviation.

**Table 4:** Confirmatory factor analysis of RKI-M.

Model	Number of items	GFI	AGFI	CFI	RMSEA	Chi-squared	df	*p*-value
4-factor	20	0.82	0.77	0.79	0.082	320.488	164	<0.001
3-factor	13	0.90	0.90	0.89	0.073	109.071	62	<0.001
2-factor	11	0.92	0.87	0.91	0.074	76.678	43	<0.001

*All factors were derived and developed from the original study of Bedregal et al. (4) to fit the Malaysian context. GFI, Goodness of Fit; AGFI, Adjusted Goodness of Fit; CFI, Confirmatory Fitness Index; RMSEA, Root Mean Square Error of Approximation; df, degree of freedom.

**Table 5:** Comparison of factor structure between the original study and this study.

Factor structure	Items based on the original study (4)	Items based on this study
Factor 1: Roles and responsibility in recovery	2, 6, 7, 9, 10, 11, 18	2, 6, 10, 11, 18
Factor 2: Non-linearity of the recovery process	4, 14, 15, 16, 17, 19	4, 14, 15, 16, 17, 19
Factor 3: The roles of self-definition and peers in recovery	1, 3, 8, 12, 20	Nil
Factor 4: Expectation regarding recovery	5,13	Nil

**Table 6:** RKI-M total mean comparison based on type of occupation (*N* = 143).

Occupation	*N*	Mean	SD
Psychiatrists	18	3.30	0.42
Medical officers	57	3.49	0.49
Medical assistants and staff nurses	56	3.97	0.54
Occupational therapists and psychologists	12	3.70	0.39

## 4. Discussion

The purpose of this study was to validate the Malay version of the RKI and translate it. This study demonstrated that the 20-item RKI-M did not exhibit good construct validity among Malaysian mental health care workers. However, a good fit was achieved when 9 items were removed, producing 2 factors based on 11 items. This was not surprising, as the authors of the original study found that during the development of the RKI, the data collected was preliminary. Moreover, there were several flaws in that study, which may explain the failure of our study to replicate the RKI entirely (4). The absence of confirmatory results is also supported by other studies’ lack of conclusive findings for the factor structure (21–23). Earlier studies in the United States and Norway showed improved construct validity when items with low Cronbach’s alpha values of <0.3 were removed. Subsequently, the best fit was found to be 1 factor loading of 10 items (24, 25). A study based on an Asian sample indicated acceptable reliability with 3 factors comprising 16 items (21). Therefore, the failure to confirm the original four-factor solution in this RKI-M is not surprising.

In our study, five out of seven items were loaded on Factor 1 (roles and responsibility) and all six items were loaded on Factor 2 (non-linearity of the recovery process), based on the original factor structure (4). These findings are consistent with several other studies that reported similar item loading on Factor 1 (21, 24, 26). Carvalho and Chima (24) found that taking a single factor structure and renaming Factor 1 as a ‘recovery process’ rather than ‘roles and responsibility’ was more inclusive of Recovery. These two studies that used the method of exploring the factors and confirming it with CFA came out with a similar construct. However, the omission of 10 questions and the existence of a potential second factor would indicate that the concept of recovery is not entirely integrated into the single factor structure (24, 25). In this aspect, the RKI-M proved to have a better outcome with a two-factor structure and the possibility of a third factor. Further research is required to elucidate hidden items to be more in keeping with local practices.

In this RKI-M study, item 1 ‘the concept of recovery’ is equally important in all ‘phases of treatment’ and item 5 ‘not everyone is capable of actively participating in the recovery process’ representing Recovery Readiness, was removed, as it had a low factor loading. Rehabilitation which is an important part of recovery and it being seen as a return to symptom-free normalcy has been challenged in the context of mental health care. People affected by mental illness have been more vocal about expressing what makes them move beyond the status of “patient” (27). This is clearly the number one point of contention between mental health professionals in providing adequate support for people with psychiatric disorders. In addition to supporting the individual and assisting them in identifying their own strengths, practitioners must be aware of the possibilities of individuals rather than concentrating solely on the problem (28). Practitioners should cease acting like experts and let people take control of their own recovery processes by letting people choose the services they want (29). Recovery is just not about services, interventions, or support, but about what people with mental disorders do to treat their condition and get their lives back on track. Consequently, recovery is not the same thing that service providers may or may not do for clients, no matter how well-intentioned or remedial they are (27). There is a misconception that recovery cannot be an “add-on” to an already-existing service, support, or system in clinical practise (30). Instead, recovery should always be the main objective of all programmes and aid, with each client having a unique recovery plan that offers a more comprehensive framework for incorporating system initiatives like evidence-based practises, cultural competency, trauma, and co-occurring conditions. To aid in recovery, a few of these factors require refocusing (31). Calls for change should, at least initially, concentrate on redesigning existing policies, practises, procedures, services, and support with an emphasis on recovery and be receptive to suggestions that include collaborative practises. More than 86% of the participants in this study came from ward settings. Thus, respondents may be defensive and prefer safety to risk-taking. This is the case in the Japanese study by Chiba et al. (21) that was conducted in an Asian setting, reflecting the similar prevailing view of prolonged hospitalization for psychiatric treatment in Malaysia (32). RKI scores in community facilities were higher than those in inpatient psychiatry, according to a follow-up study by Chiba (33), despite conflicting results from earlier studies (6, 34). Additional studies show the challenges in providing recovery-oriented care in hospitals (35, 36). As a result, the higher RKI scores found among those with community support can be explained by the fact that these individuals are typically more exposed to social resources that promote personal recovery and have access to a greater number of individuals who have successfully completed their own recovery (37). This demonstrates how crucial it is to have a knowledgeable community psychiatry team.

Historically, Malaysia has gone through several phases of development of community psychiatric services and decentralization of services outside psychiatric hospitals since the 1970s (32). The results showed questions 13 and 20 had a low factor loading ratio, which may be attributed to the low domain of recovery knowledge of the mental health practitioners in this study. Lack of local data to support this hypothesis, reflects the lack of epidemiological studies in Malaysia that looks at mental health literacy and help-seeking behavior. Understanding of mental health and seeking help may have improved in recent years as the Malaysian media openly shares and discusses mental health issues. However, a systematic epidemiological investigation is still needed to prove this situation (38). This is undoubtedly the first point of contention among mental health professionals when it comes to giving people with psychiatric disabilities adequate support. Instead of focusing on the issue, practitioners need to be aware of everyone’s options (39). Additionally, they must encourage and assist people in discovering their own strengths (28). Recovery refers to what people with mental disorders do to treat their condition and restore control of their lives, not to any service, intervention, or support. Therefore, regardless of how well-intentioned or recovery-oriented service providers may be, recovery does not equal something that service providers may or may not do for clients (27). At least initially, the focus of transformation should be on modifying and realigning current policies, practises, services, and support to be focused on promoting recovery, enacting collaborative practises, and being receptive to the idea of applying the Recovery-oriented approach’ tenets.

The RKI measures risk, and among the questions it poses are those that inquire about the clinician’s viewpoint on whether risk-taking should be encouraged to achieve recovery or prevented (4). Even though hope has been included in the original concept of recovery, most health care workers struggle to translate it into support for our clients, as being hopeful involves empowering clients to take therapeutic risk. Hope is mainly investigated as mechanisms related to health and quality of life that create everyday possibilities (40–42). Unfortunately, many clients experience daunting and discouraging interactions with mental health professionals due to their low expectations, which destroy hope (34). Professionals need to understand and strive to promote hope because it is these interpersonal relationships that serve as a catalyst for hope, which is critical to recovery (34). Lack of organizational support, exhaustion, burnout (43), the absence of a therapeutic relationship (44) and working with service users whose needs are complex and progress is slow (45) may all contribute to practitioners’ lack of optimism (46). The literature suggests several approaches to resolving this problem, including clinical supervision for all employees and encouragement of therapeutic alliances through instruction on how to practise recovery (47). These factors may be why recovery approaches in Malaysia are hampered by a lack of resources, understanding and communication between fraternities, although we have moved to community-oriented practices since the 1970s.

One of the most important concepts in the recovery approach is the individual process and shared decision-making (SDM). Previous research in Malaysia has indicated that SDM is one of the focal points in the individual process. Although this idea is present in Malaysia, it is still in its early stages (48). In this RKI-M study, the findings show that most mental health care workers had poor knowledge on the individual process. This fact was picked up by item 7 which had a low factor loading, indicating that the knowledge of this aspect was still poor and insufficient. With the recent adoption of the National Health Plan, there are opportunities to promote SDM among the Ministry of Health, public and private health service providers, researchers, academic institutions, and to involve patients in health care decision making (48).

In order to provide the highest level of care, the World Health Organization (WHO) defines collaborative practise as multiple health professionals from diverse professional backgrounds working with patients, families, carers, and communities (49). A multidisciplinary team’s members working together as well as patient and healthcare professional collaboration are both examples of broad collaboration. Due to the complexity of serious mental health issues, effective care is typically team-based, with many different specialties collaborating to assist clients while keeping in mind the preferences of service users. It is considered important to develop cooperative practices in Malaysia, as qualitative studies have shown that interactions are often hierarchical rather than supportive. This scenario sometimes negatively affects patient care, for example, when the nurse does not inform the doctor in charge that the treatment plan is not working or that patient has stopped taking the drug because of side effects. For example, services are often fragmented because there is little exchange of information between psychiatric hospitals and health clinics. Even in primary care, there is a treatment gap of over 90%. Cultural factors influence how people work together, and caregiving models are typically developed in Western settings, so they may not be the best caregiving models for Asian environments, rendering poor representations (50).

This study revealed an intriguing finding: Staff nurses, medical assistants, and other members of the allied health services seemed to know more about recovery than psychiatrists and medical officers, who had a lower total mean knowledge score. Perhaps this result could be explained by the two different approaches used in the undergraduate training modules that either use the recovery model or the medical model. Randomized controlled trials have shown that mental health user-trained professionals have higher rates of positive recovery than expert-trained professionals (51). Other researchers have discovered that recovery training programmes could indeed change practitioners’ attitudes, knowledge, and hopefulness (52–54). A study done among nurses in Italy, shows that gender and age to play a role in recovery knowledge and its implementation (55). It will be interesting to do this in future studies among student nurses as well as to analyze their age and gender and seeking out if indeed current syllabus and age does affect recovery knowledge.

The World Health Organization (WHO) observes the development of mental health as an important task in the coming years. WHO uses several strategies to achieve this goal and one of them is the promotion of Recovery-orientated treatment (56). Despite the limitations brought on by the illness, the emphasis is on leading a fulfilling, hopeful life and being able to contribute to society (47). There are two popular types of recovery: “Personal recovery,” which is typically said to be based on the experiences of people living with mental illness; and “clinical recovery,” which derives from the knowledge of mental health professionals and includes symptom relief, restoration of social functioning, and support for patients to “get back to normal” (57). Organizational commitment, individualized recovery, and positive working relationships are essential elements (58) however, medical treatment typically take center stage in acute care facilities. Psychosocial interventions may be less frequently used in these settings because of the impact this medical focus has on mental health professionals working there (59–63). Health professionals often take a symptom-focused approach to mental health care, which can undermine the development and implementation of Recovery practices (64).

The concept of ‘recovery’ developed in Western countries differs from that developed in Asian countries and in English-speaking countries (65) leading to only the first two factors to be being similar to Bedregal et al.’s (4) the original RKI study by Bedregal et al. (4), and the and possibility of poor recovery knowledge among the Malaysian mental health care workers. The cultural context is also important when examining mental health beliefs. Cultural differences by race have led to different definitions of mental health (66). An important part of mental health in Malaysian culture has to do with spiritual and religious factors (67). Malays associate mental illness with the term’s “madness” or “gila” or “sakit jiva” (disease of the soul). Mental disorders are generally considered paranormal rather than clinically confirmed symptoms. The general perception of mental illness is typically expressed because of rejecting or ignoring traditional values, so Malaysian culture itself has a significant impact on society (68). The majority ethnic group in Malaysia, the Malays, believe that mental illness has a supernatural origin, which is a form of divine punishment, or is the result of excessive mental effort (69). Similar to Malay culture, traditional Chinese medicine, which is based on Confucianism and Taoism, has an influence on how the Chinese view mental health (70). The majority of Chinese believe that an imbalance between yin and yang can result in mental illness, and these principles are related to the idea of yin and yang as a symbol of life (70). Hinduism’s view of mental health is based on the idea that the four purposes of life—Dharma, Karma, Artha, and Moksha—are reflected in a mind–body dichotomy. Hindus believe that these four components are out of balance in those who suffer from mental illness (71). Cultures and religions have a significant impact on culturally sensitive aspects of religions and belief systems. The use of traditional treatments for mental illness in society may be to blame for this predicament. Mental health professionals, who make up a sizable portion of the community, are plagued by the stigma of public political hype about mental health in their day-to-day work. Hence, future studies ought to concentrate on the cultural specificity of recovery attitudes and information.

There are very few published recovery-intervention studies using validated tools and training programmes (72). However, there are frequently discussed interdisciplinary approaches to community care that are published. For instance, Slade et al. multi-site randomized controlled trial in 2015 that investigated behaviorally focused interventions by mental health team members to enhance recovery support for people with mental illness (REFOCUS) (73). To this date, no recovery-intervention study has still not been done in Malaysia and little efforts taken to standardize quality measures of mental health care locally and around the world. In addition to enabling quality improvement at the provider, clinic, and health system levels, systematic measurement and reporting of healthcare quality also makes it possible for accountability mechanisms like financial penalties, public accountability, and compensation (74). It is challenging to evaluate the quality of mental health care globally because it varies from one nation to another and from one service provider to another (75). Hence, it is not only important to define outcome measures but to have consistent outcome measures first before being able to conduct valid and actionable studies. Existing e-health systems lack the ability to systematically collect data, which can hinder continuous improvement in patient quality (75). To ease this problem, mental health professionals recommend the systematic use of outcome measures using intervention-based therapy. The weak infrastructure within health systems here in Malaysia, make it complicated to have measured outcomes, and this is especially true given the numerous barriers to mental health, including policy and technological limitations, and limited scientific evidence on qualitative measures of mental health. There is inadequate training and support for healthcare providers and cultural barriers to integrating mental health services into the general healthcare environment. There are many gaps in the scientific basis for supporting mental health quality measures, particularly for consumers, as well as for the most meaningful outcomes for specific population groups, such as children. There is a lack of resources to detect and measure common psychiatric disorders in the population, such as anxiety disorders or even outcomes of evidence-based treatments such as psychotherapy. The evidence base for many other psychosocial interventions is still lacking, even though there is a well-established evidence base for mental health interventions like drug therapy, specialized passive psychotherapy (like cognitive behavioral therapy), and team-based interventions (like community mindfulness therapy) (76). Evidence-based psychotherapy is currently subject to quality standards that may not accurately reflect how well it is delivered.

Collaboration in client treatment has been hampered by varying definitions of recovery. Research on the recovery model has produced a complex definition over time with no apparent agreement. Battersby and Morrow (61) conducted a conceptual analysis and discovered that different disciplines, including social work, nursing, and psychology, have different definitions of recovery. However, according to literature review, quality of life, self-determination, empowerment, hope, meaningful roles, peripheral effects of serious mental illness, support system, and distinctive treatment are among the factors that define recovery (77–82). To streamline the dissemination of knowledge and research on the recovery model, researchers looked at the various definitions of recovery that are currently in use and identified recurring themes (77, 83, 84). In this study we have attempted to explore the Malaysian understandings of Recovery knowledge and to extrapolate the local influences. Nevertheless, action has been hampered by a lack of agreement on what constitutes operational and measurable recovery among healthcare professionals, the research community, and most crucially, mental health consumers (85). It’s not always necessary to expect self-report scales to have high internal consistency. This is due to the fact that individuals are knowledgeable in some fields but not in others (21). Therefore, the low reliability observed in this study seems to be understandable because the RKI is a measure for assessing human knowledge. The 11 RKI-M items’ validity and reliability are somewhat supported by this study, but modifications will be needed in follow-up research. Wilrycx et al. (23) reported that the organization and presentation of RKI entries is complicated and challenging to interpret. Following careful consideration of RKI-M representations and conceptual equivalence, it allows for a clearer conceptualization of “retrieval knowledge and retrieval relationships” in the context of the original RKI.

## 5. Limitations

There are some limitations on this study. First, test–retest reliability was not evaluated. Second, registered nurses, medical assistants, and psychiatry medical officers made up 75% of the total study population. Consequently, the generalizability of our findings might only apply to these professions. AGFI was a borderline good fit as AMOS requires a minimum sample size of 300. Third, the English language, when translated into Bahasa Malaysia, could prove to have low-level comprehension and may not be very suitable for allied health professionals, as the words used are more formal and less intricate. There is also a more specific need to understand the concept of the recovery approach before one can complete the questionnaire. Differences could have arisen because of the translation of the items from English to Bahasa Malaysia. During the translation process, issues arose, for instance, finding equivalent words in Bahasa was difficult for some items that were just extremely difficult to understand. A simpler-worded Bahasa questionnaire would be better and could be used for both professional health care workers and allied health workers. Fourth, differences may arise because of the way mental health care is organized in Malaysia. For instance, the multi-cultural society is unfamiliar with consumer-run initiatives, particular recovery tenets, managed care, or collaborating with individuals who have personally dealt with psychiatric issues.

## 6. Conclusion

This study investigated the factor validity and internal consistency of the Bahasa Malaysia version of the RKI among mental health professionals. The 20-item RKI-M was reliable but had poor construct validity. However, the modified 11-item-Malay version of RKI is a more reliable measure and had with a good construct validity. Malaysian cultural settings influenced the two-factor structure in the present study. Omitted items do not consistently measure the same concept in the definition of recovery. CFA found a third factor, but there were insufficient entries for this factor, resulting in a low Cronbach’s alpha. This result may indicate the possibility of a hidden factor. The authors humbly recommend the use of both models in future studies. EFA should be performed to find the factors in the first sample, then CFA should be applied to the second sample. The current scale can be used for future studies in Malaysia, but future large-scale studies are needed for reliable validation.

## Data availability statement

The raw data supporting the conclusions of this article will be made available by the authors, without undue reservation.

## Ethics statement

The studies involving human participants were reviewed and approved by Medical Research and Ethics Committee, National University of Malaysia. The patients/participants provided their written informed consent to participate in this study.

## Author contributions

TM, SL, FA, and MM conceptualized this study. SL was involved in the database collection and organization of this study. KT and SL and were responsible for data analysis. SL, TM, FA, KT, and MM were responsible for interpretation of study results and involved in the writing and review of the final draft of this manuscript. All authors contributed to the article and approved the submitted version.

## Conflict of interest

The study’s authors affirm that there were no financial or commercial ties that might be viewed as having a potential conflict of interest.

## Publisher’s note

All claims expressed in this article are solely those of the authors and do not necessarily represent those of their affiliated organizations, or those of the publisher, the editors and the reviewers. Any product that may be evaluated in this article, or claim that may be made by its manufacturer, is not guaranteed or endorsed by the publisher.
